# Development and characterization of EST‐SSR markers for *Saxifraga fortunei* var. *incisolobata* (Saxifragaceae)

**DOI:** 10.1002/aps3.11275

**Published:** 2019-07-09

**Authors:** Kana Magota, Daiki Takahashi, Hiroaki Setoguchi

**Affiliations:** ^1^ Graduate School of Human and Environmental Studies Kyoto University Yoshida Nihonmatsu‐cho, Sakyo‐ku Kyoto 606–8501 Japan

**Keywords:** ecological polymorphisms, expressed sequence tag–simple sequence repeat (EST‐SSR) markers, *Saxifraga fortunei*, Saxifragaceae, transcriptome

## Abstract

**Premise:**

*Saxifraga fortunei* (Saxifragaceae) includes several infraspecific taxa that are ecologically and morphologically distinct. To investigate the evolutionary history of phenotypic polymorphisms in this species, we developed expressed sequence tag–simple sequence repeat (EST‐SSR) markers for *S*. *fortunei*.

**Methods and Results:**

We developed 26 polymorphic markers based on transcriptome data obtained from Illumina HiSeq 2000. Within three populations of *S*. *fortunei* var. *incisolobata*, the number of alleles ranged from four to 25, and the levels of observed and expected heterozygosity ranged from 0.200 to 0.847 and from 0.209 to 0.930, respectively. Furthermore, all 26 loci showed transferability for *S*. *fortunei* var. *obtusocuneata* and *S*. *fortunei* var. *suwoensis*, and 18 loci were also successfully amplified in *S*. *acerifolia*.

**Conclusions:**

These newly developed EST‐SSR markers will prove useful to infer the evolutionary history of *S*. *fortunei* var. *incisolobata* and its relatives in population genetic studies.

*Saxifraga* L. is the largest genus in the Saxifragaceae family, with more than 440 species widely distributed throughout the Northern Hemisphere (Tkach et al., 2015). A recent phylogenetic study divided this genus into 13 sections and nine subsections, with section *Irregulares* Haw., a well characterized group with zygomorphic flowers, as the earliest diverged lineage (Tkach et al., 2015). This section comprises 15–20 species growing in moist temperate areas of East Asia, whereas most other sections of *Saxifraga* are widely distributed in boreal and/or alpine areas (Pan, 2001).

*Saxifraga fortunei* Hook., which belongs to sect. *Irregulares*, is a perennial herb distributed in East Asia, ranging from mainland China to Sakhalin and throughout the Japanese Archipelago. This species is ecologically and morphologically diverse and includes more than seven infraspecific ecotypic entities (Nakai, 1938; Ohba, 1982; Pan, 2001). *Saxifraga fortunei* var. *incisolobata* (Engl. & Irmsch.) Nakai is the most widely distributed taxon, growing in shaded understory. *Saxifraga fortunei* var. *obtusocuneata* (Makino) Nakai is a riparian taxon with a cuneate leaf blade base, and *S*. *fortunei* var. *suwoensis* Nakai is also a riverbank taxon with deeply dissected leaf blades, and these two taxa have allopatric distributions in western Japan. There are other local endemics with specific characters, such as an alpine taxon that grows under direct sunlight, and an insular taxon with thick and deeply haired leaf blades. These intraspecific taxa are presumably adapted to specific habitats, and these patterns of phenotypic variation provide an ideal model for the investigation of ecological adaptation and diversification. Magota et al. (2018) reported several chloroplast and nuclear microsatellite markers based on genomic DNA sequence data of *S*. *acerifolia* Wakabayashi & Satomi, an endangered plant species related to *S*. *fortunei* (Wakabayashi, 1973; Ministry of the Environment, Japan, 2019). However, only two of the previously identified nuclear markers showed polymorphisms in *S*. *fortunei*. Therefore, more polymorphic markers were needed to investigate the genetic structure and to infer the evolutionary history of *S*. *fortunei*. Expressed sequence tag–simple sequence repeat (EST‐SSR) markers are valuable for their cross‐transferability to related taxa in many plant species, and they are easier to develop at a lower cost than other types of nuclear markers (e.g., Takahashi et al., 2017). In this study, we developed EST‐SSR markers of *S*. *fortunei* var. *incisolobata* and examined their utility and transferability to related taxa.

## METHODS AND RESULTS

Fresh floral buds of *S*. *fortunei* var. *incisolobata* (population F42, Appendix 1) were frozen in liquid nitrogen and total RNA was extracted using the Agilent Plant RNA Isolation Mini Kit (Agilent Technologies, Santa Clara, California, USA) following the manufacturer's protocol. A cDNA library was constructed and sequenced using the Illumina HiSeq 2000 platform (Illumina, San Diego, California, USA; performed by BGI, Shenzhen, China). The raw reads (paired‐end 100 bp) are deposited in the DNA Data Bank of Japan (DDBJ; BioProject PRJDB8004). Low‐quality reads were trimmed using Trimmomatic 0.38 (Bolger et al., 2014) with the following parameters: HEADCROP, 20 and SLIDINGWINDOW, 4:20. In all, 26,177,799 paired reads were obtained. We conducted de novo transcriptome assembly of these reads using Trinity v.2.8.4 (Haas et al., 2013), which produced 121,463 contigs (mean length 673 bp). Microsatellite regions (≥7 dinucleotide or ≥7 trinucleotide repeats) were screened using MSATCOMMANDER (Faircloth, 2008). A total of 568 regions were obtained, and we selected 96 PCR primer pairs based on the repeat numbers of microsatellite motifs. For all loci, the forward primers were synthesized with one of four different M13 sequences (5′‐CACGACGTTGTAAAACGAC‐3′, 5′‐TGTGGAATTGTGAGCGG‐3′, 5′‐CTATAGGGCACGCGTGGT‐3′, or 5′‐CGGAGAGCCGAGAGGTG‐3′), and the reverse primers were tagged with a PIG‐tail sequence (5′‐GTTTCTT‐3′).

Twenty‐four *S*. *fortunei* var. *incisolobata* individuals from each of three populations (F05, F35, and F38; Appendix 1) were used to evaluate the polymorphisms of the target loci. Moreover, we used 24 individuals from each of three related taxa (*S*. *fortunei* var. *obtusocuneata*,* S*. *fortunei* var. *suwoensis*, and *S*. *acerifolia*) for cross‐amplification. Genomic DNA for PCR was extracted from dried leaf materials using the cetyltrimethylammonium bromide (CTAB) method (Doyle and Doyle, 1987), after washing the leaf powder three times with HEPES buffer (pH = 8.0; Setoguchi and Ohba, 1995). The PCR was performed in a 5‐μL reaction volume, containing approximately 0.5 ng DNA, 2.5 μL 2× Multiplex PCR Master Mix (QIAGEN, Hilden, Germany), 0.01 μM forward primer, 0.2 μM reverse primer, and 0.1 μM fluorescence‐labeled M13 primer. The PCR thermal profile was set as follows: an initial denaturation at 95°C for 30 min; followed by 35 cycles of 95°C for 30 s, 58°C for 3 min, and 68°C for 1 min; and then a final extension at 68°C for 20 min. Amplified products were loaded onto an ABI 3130xl Genetic Analyzer (Applied Biosystems, Carlsbad, California, USA) using the GeneScan 600 LIZ Size Standard (Applied Biosystems), POP7 polymer (Applied Biosystems), and a 36‐cm capillary array. Fragment size was determined using GeneMapper software (Applied Biosystems). To evaluate the utility of the developed markers, genetic diversity indices (number of alleles, observed heterozygosity, and expected heterozygosity) were calculated using GenAlEx version 6.503 (Peakall and Smouse, 2006). Significant deviations from Hardy–Weinberg equilibrium and linkage disequilibrium were tested with 1000 randomizations using GENEPOP 4.2 (Raymond, 1995).

Of 96 primer pairs tested with an individual from population F42, 47 loci showed clear peaks. Of the 47 loci that were successfully amplified, 26 showed polymorphisms within each population of *S*. *fortunei* var. *incisolobata* (Table 1) and 21 were monomorphic (Appendix 2). In total, from three populations (F05, F35, and F38), the number of alleles ranged from four to 25 and the levels of observed and expected heterozygosity ranged from 0.200 to 0.847 and from 0.209 to 0.930, respectively (Table 2). In all three populations, two loci (SF716 and SF314) significantly deviated from Hardy–Weinberg equilibrium (*P* < 0.01), and significant linkage disequilibrium was detected between loci SF716 and SF166 (*P* = 0.00951).

**Table 1:** Characteristics of 26 polymorphic microsatellite loci developed for *Saxifraga fortunei* var. *incisolobata*.[Link]

Locus	Primer sequences (5′–3′)	Repeat motif	Allele size range (bp)	BLASTX top hit description	*E*‐value	GenBank accession no.
SF1037	F: CGGAGAGCCGAGAGGTGCAGTTGCTCACCACAAGACC	(GAA)_8_	429–445	PREDICTED: polynucleotide 3′‐phosphatase ZDP [*Vitis vinifera*]	6.08E‐161	LC465769
	R: GTTTCTTCTTCGTCTTCCTTGCGAACC					
SF1424	F: CGGAGAGCCGAGAGGTGAAGCCACATTCCTTTCCACC	(CCA)_11_	329–350	Probable beta‐D‐xylosidase 2 isoform X1 [*Olea europaea* var. *sylvestris*]	0.00	LC465771
	R: GTTTCTTATGAAGAGCCTCAGACCACC					
SF816	F: CGGAGAGCCGAGAGGTGCCCACATTCCTGGCATTGTG	(GAT)_9_	317–344	Uncharacterized protein LOC18994464 [*Eutrema salsugineum*]	4.08E‐24	LC465773
	R: GTTTCTTAAAGAACAAACATAGCCACGAC					
SF1057	F: CTATAGGGCACGCGTGGTGACTTCCCATAGCTCCTCCG	(CT)_11_	303–335	No significant hit	—	LC465774
	R: GTTTCTTGGCGATCAGAACCCAACAATC					
SF75	F: TGTGGAATTGTGAGCGGATGGGACCAGCAGCATAAGG	(AGG)_8_	340–359	Ubiquitin carboxyl‐terminal hydrolase 8 [*Ziziphus jujuba*]	9.77E‐07	LC465775
	R: GTTTCTTCGGAAGATCTGCATCACGTC					
SF1016	F: CTATAGGGCACGCGTGGTTCTGTGGAAACCTCACTTCTTG	(AAG)_8_	252–279	Uncharacterized protein LOC102620652 [*Citrus sinensis*]	0.00	LC465776
	R: GTTTCTTCTGGTTCTCGTCACAAACCG					
SF166	F: TGTGGAATTGTGAGCGGATGGTGGTGGTGATGACAAG	(GA)_12_	221–234	No significant hit	—	LC465777
	R: GTTTCTTGCGCATCTTCCTTCTCTCAAC					
SF143	F: CACGACGTTGTAAAACGACCCTCGACATCAAGGTTCACAC	(CT)_12_	218–266	PREDICTED: uncharacterized protein LOC100256691 [*Vitis vinifera*]	0.00	LC465780
	R: GTTTCTTATTTGGTTCCTTGCGTGTCC					
SF716	F: CACGACGTTGTAAAACGACGAAGCCTTGAGTTGATTTCGC	(ATT)_8_	191–230	No significant hit	—	LC465782
	R: GTTTCTTTTCAGGCCTCCCATCACATG					
SF319	F: TGTGGAATTGTGAGCGGCGGAGGTTGAGATTGAAGGC	(TC)_10_	361–397	Uncharacterized protein LOC110815042 [*Carica papaya*]	3.75E‐15	LC465783
	R: GTTTCTTTTACCAAACGGCCAGCATTC					
SF1102	F: CTATAGGGCACGCGTGGTCTCTTCTATCTCCTCGGCCG	(ATC)_7_	195–207	Uncharacterized protein DDB_G0290685‐like [*Quercus suber*]	2.57E‐06	LC465784
	R: GTTTCTTTGGCATGTCAAAGCCATCTG					
SF479	F: TGTGGAATTGTGAGCGGGGAGATCCGCATGAAACACG	(GAT)_8_	162–189	PREDICTED: uncharacterized protein LOC104591093 [*Nelumbo nucifera*]	2.97E‐13	LC465786
	R: GTTTCTTTCTATAAACGGCGATGAGTTGG					
SF314	F: CGGAGAGCCGAGAGGTGGTGGTGTAGAAGGGTGAGGG	(GTG)_9_	124–176	PREDICTED: protein CURVATURE THYLAKOID 1D, chloroplastic [*Vitis vinifera*]	1.24E‐60	LC465788
	R: GTTTCTTCAAAGCCTCTCCTATGGTGC					
SF385	F: TGTGGAATTGTGAGCGGACAGGAGGTGGTTTGTAGGG	(GGT)_8_	105–186	No significant hit	—	LC465790
	R: GTTTCTTGCCTTCACCTTCTCCACCC					
SF1135	F: CTATAGGGCACGCGTGGTCATATTGCCTCGCTGTCCAG	(CT)_13_	129–141	No significant hit	—	LC465791
	R: GTTTCTTTGTGTTGGATTACGTGGGTG					
SF1450	F: CGGAGAGCCGAGAGGTGAGGCGCCGATTTGTTTGTC	(GA)_12_	119–133	WD40 repeat‐containing protein HOS15 [*Momordica charantia*]	0.00	LC465792
	R: GTTTCTTTTTCCCGTCACATCCGTACC					
SF941	F: CACGACGTTGTAAAACGACGATCCGGCAACTGTTCAAGG	(TGG)_7_	361–463	Hypothetical protein CDL15_Pgr014496 [*Punica granatum*]	1.39E‐40	LC465793
	R: GTTTCTTACTTTCTTGCAACTTCAACAGC					
SF1144	F: TGTGGAATTGTGAGCGGGCCGAAGTAACAACACCACC	(TGC)_7_	395–479	DEAD‐box ATP‐dependent RNA helicase 3, chloroplastic [*Vitis vinifera*]	0.00	LC465794
	R: GTTTCTTAGAGAGAGGTGGAAGTGTGC					
SF1529	F: TGTGGAATTGTGAGCGGAGCTCTAAGAAACGGCGAAAC	(ATC)_7_	317–365	Homeobox‐leucine zipper protein ATHB‐13 [*Ricinus communis*]	7.32E‐75	LC465799
	R: GTTTCTTGATGTTGCTCTGTCCATGGC					
SF1116	F: CGGAGAGCCGAGAGGTGAATGGCGGCAGTTTACTTGC	(AAC)_7_	275–304	PREDICTED: proliferating cell nuclear antigen [*Daucus carota* subsp. *sativus*]	2.50E‐95	LC465804
	R: GTTTCTTAAGTTTGCGTCGTTCACCAG					
SF1222	F: CACGACGTTGTAAAACGACCTGGTCAGAGAGTGTGGAGG	(GAT)_7_	246–270	Unnamed protein product, partial [*Vitis vinifera*]	2.34E‐136	LC465805
	R: GTTTCTTCTCCAGAAACCCTAGGCTCC					
SF489	F: CGGAGAGCCGAGAGGTGAAACTCACTTCGCCATGTGC	(CT)_9_	363–371	Unnamed protein product, partial [*Vitis vinifera*]	1.73E‐87	LC465807
	R: GTTTCTTTCCAGACGCCAGTTTCTCAC					
SF644	F: CACGACGTTGTAAAACGACAATTGCCCGGTTGATGCATC	(AG)_10_	168–210	No significant hit	—	LC465813
	R: GTTTCTTCCCTACCAACAAAGTCGTACC					
SF664	F: CGGAGAGCCGAGAGGTGTCTTACTGCCCAGAACTCCAG	(AAT)_7_	145–171	Zinc finger CCCH domain‐containing protein 53 isoform X1 [*Glycine max*]	2.08E‐172	LC465814
	R: GTTTCTTAATCACTCACACGGGAATACTC					
SF631	F: CACGACGTTGTAAAACGACACTGAACAGATCTCCATGGC	(TA)_9_	143–171	No significant hit	—	LC465815
	R: GTTTCTTTGCACCATACTTACGAGGCC					
SF519	F: CTATAGGGCACGCGTGGTCACTCCCATGAACCTACCAAG	(AAT)_7_	111–129	Ferredoxin‐3, chloroplastic [*Vitis vinifera*]	1.44E‐83	LC465816
	R: GTTTCTTTCACACACACAAGGAAAGCG					

Annealing temperature is 58°C for all primer pairs.

**Table 2:** Genetic diversity statistics for three populations of *Saxifraga fortunei* var. *incisolobata* based on 26 newly developed EST‐SSR markers.^a^

Locus	F05 (*N* = 24)			F35 (*N* = 24)			F38 (*N* = 24)			Total (*N* = 72)		
	*A*	*H*_o_ b	*H*_e_	*A*	*H*_o_ b	*H*_e_	*A*	*H*_o_ b	*H*_e_	*A*	*H*_o_	*H*_e_
SF1037	4	0.455	0.415	7	0.778	0.769	7	0.667	0.675	8	0.625	0.718
SF1424	7	0.583	0.732	8	0.917	0.793	9	0.750	0.801	10	0.750	0.815
SF816	7	0.750	0.828	8	0.696	0.841	9	0.833	0.854	10	0.761	0.861
SF1057	13	0.792	0.885	11	0.875	0.855	14	0.875	0.890	17	0.847	0.899
SF75	3	0.391	0.373	3	0.095	0.092	4	0.208	0.261	7	0.235	0.265
SF1016	6	0.583	0.564	8	0.833	0.834	8	0.667*	0.780	12	0.694	0.811
SF166	11	0.417*	0.615	10	0.682	0.808	9	0.583**	0.829	15	0.557	0.783
SF143	10	0.714	0.883	12	0.650*	0.871	20	0.875	0.920	22	0.754	0.930
SF716	10	0.391***	0.836	9	0.391***	0.780	9	0.458**	0.773	15	0.414	0.834
SF319	9	0.542**	0.865	13	0.818	0.882	11	0.750	0.852	15	0.700	0.888
SF1102	4	0.333**	0.637	4	0.636	0.657	5	0.333**	0.607	5	0.429	0.687
SF479	4	0.583	0.559	7	0.583	0.707	10	0.875	0.852	10	0.681	0.787
SF314	7	0.375***	0.680	6	0.375***	0.707	11	0.409***	0.854	14	0.386	0.787
SF385	16	0.609***	0.869	14	0.714	0.897	14	0.500***	0.884	25	0.603	0.925
SF1135	3	0.208	0.254	6	0.292	0.387	3	0.583	0.424	7	0.361	0.375
SF1450	5	0.500	0.617	7	0.545	0.727	9	0.625*	0.788	10	0.559	0.742
SF941	8	0.087***	0.767	5	0.318**	0.629	6	0.458	0.574	10	0.290	0.751
SF1144	8	0.739	0.781	12	0.783	0.830	10	0.833	0.846	16	0.786	0.845
SF1529	6	0.500	0.549	6	0.875	0.722	9	0.708	0.809	9	0.694	0.751
SF1116	2	0.048	0.046	5	0.250*	0.448	6	0.292	0.362	8	0.200	0.304
SF1222	3	0.125	0.119	4	0.174	0.164	4	0.375	0.325	7	0.225	0.209
SF489	2	0.042	0.041	4	0.696	0.695	4	0.375*	0.574	4	0.366	0.509
SF644	9	0.750	0.724	16	0.833	0.866	11	0.708	0.759	20	0.764	0.825
SF664	3	0.292	0.254	3	0.217	0.198	3	0.375	0.398	4	0.296	0.296
SF631	10	0.458***	0.823	10	0.609**	0.852	10	0.708	0.790	13	0.592	0.864
SF519	5	0.625	0.734	4	0.652	0.593	7	0.792	0.734	8	0.690	0.725
Average	6.7	0.458	0.603	7.6	0.588	0.677	8.5	0.616	0.701	11.6	0.548	0.700

Note

*A* = number of alleles; *H*_e_ = expected heterozygosity; *H*_o_ = observed heterozygosity; *N* = number of individuals sampled.

a Locality and voucher information are provided in Appendix 1.

b Asterisks indicate significant deviation from Hardy–Weinberg equilibrium after Bonferroni correction (*P < 0.05, **P < 0.01, ***P < 0.001).

The results of cross‐amplifications are shown in Table 3. In *S*. *fortunei* var. *obtusocuneata*, all 26 loci were successfully amplified and polymorphic. In *S*. *fortunei* var. *suwoensis*, all 26 loci were amplified, of which 22 showed polymorphisms. In *S*. *acerifolia*, 18 loci were amplified and 15 showed polymorphisms.

**Table 3:** Cross‐amplification and genetic diversity statistics of EST‐SSR markers developed for *Saxifraga fortunei* var. *incisolobata* in related taxa.^a^

Locus	*S. fortunei* var. *obtusocuneata* (*N* = 24)			*S. fortunei* var. *suwoensis* (*N* = 24)			*S. acerifolia* (*N* = 24)		
	*A*	*H*_o_ b	*H*_e_	*A*	*H*_o_ b	*H*_e_	*A*	*H*_o_ b	*H*_e_
SF1037	4	0.650	0.563	2	0.125	0.117	3	0.091*	0.206
SF1424	3	0.348	0.396	3	0.083	0.081	4	0.091*	0.170
SF816	5	0.739	0.707	6	0.833	0.799	—	—	—
SF1057	6	0.565	0.676	4	0.250	0.261	3	0.091	0.088
SF75	2	0.105	0.100	3	0.435	0.468	2	0.048	0.046
SF1016	3	0.565	0.662	4	0.478	0.472	—	—	—
SF166	5	0.739*	0.662	5	0.667	0.635	—	—	—
SF143	5	0.750	0.744	4	0.583	0.622	1	0.000	0.000
SF716	8	0.864	0.846	5	0.773	0.743	6	0.105***	0.402
SF319	6	0.583	0.642	3	0.455	0.430	3	0.056***	0.545
SF1102	4	0.458*	0.548	2	0.542	0.457	—	—	—
SF479	3	0.261	0.334	2	0.083	0.080	6	0.304	0.345
SF314	6	0.708	0.787	11	0.542***	0.826	3	0.125	0.119
SF385	6	0.333***	0.549	9	0.500***	0.788	4	0.000***	0.458
SF1135	3	0.042*	0.119	3	0.565**	0.494	7	0.667	0.685
SF1450	5	0.455	0.645	2	0.042	0.041	8	0.542	0.578
SF941	5	0.542	0.739	1	0.000	0.000	—	—	—
SF1144	6	0.636	0.760	3	0.625	0.551	—	—	—
SF1529	7	0.864	0.746	4	0.609	0.632	1	0.000	0.000
SF1116	5	0.278***	0.660	1	0.000	0.000	6	0.167***	0.608
SF1222	3	0.591	0.574	2	0.042	0.041	—	—	—
SF489	4	0.542*	0.556	3	0.667	0.635	2	0.043	0.043
SF644	7	0.542	0.753	7	0.625*	0.791	16	0.696**	0.881
SF664	4	0.652	0.578	1	0.000	0.000	10	0.762	0.621
SF631	5	0.188***	0.658	1	0.000	0.000	1	0.000	0.000
SF519	3	0.478	0.532	2	0.458	0.430	—	—	—
Average	4.7	0.518	0.598	3.6	0.384	0.400	4.8	0.210	0.322

Note

*A* = number of alleles; *H*_e_ = expected heterozygosity; *H*_o_ = observed heterozygosity; *N* = number of individuals sampled.

a Locality and voucher information are provided in Appendix 1.

b Asterisks indicate significant deviation from Hardy–Weinberg equilibrium after Bonferroni correction (*P < 0.05, **P < 0.01, ***P < 0.001).

## CONCLUSIONS

We developed 26 novel polymorphic EST‐SSR markers for *S*. *fortunei* var. *incisolobata*. All loci were amplified in other infraspecific taxa of *S*. *fortunei*, and 18 of them were transferable to *S*. *acerifolia*. These markers will be useful for future studies to investigate the evolutionary histories of these species. In addition, they have also proved helpful in evaluating genetic diversity in *S*. *acerifolia*, an endangered species.

## AUTHOR CONTRIBUTIONS

K.M. and H.S. conceived and designed the experiments. K.M., D.T., and H.S. contributed to sample collection. K.M. and D.T. conducted de novo transcriptome assembly. K.M. performed the molecular laboratory work, allele scoring, and analyses. K.M. drafted the manuscript and all authors participated in manuscript modifications and approved the final version for publication.

## Data Availability

Raw reads from the cDNA library sequenced by Illumina HiSeq 2000 have been deposited to the DNA Data Bank of Japan (DDBJ; BioProject PRJDB8004). Sequence information for the developed primers has been deposited to the National Center for Biotechnology Information (NCBI); GenBank accession numbers are shown in Table 1 and Appendix 2.

## Sample information for *Saxifraga* species used in this study.

**Table:**

Taxa	Population	*N*	Collection locality	Geographic coordinates	Voucher specimen accession no.[Link]
*Saxifraga fortunei* Hook. var. *incisolobata* (Engl. & Irmsch.) Nakai	F42	1	Takahama‐cho, Ohi‐gun, Fukui Pref., Japan	35°30′N, 135°29′E	KYO_00025612
*Saxifraga fortunei* var. *incisolobata*	F05	24	Oga City, Akita Pref., Japan	39°53′N, 139°45′E	KYO_00025344
*Saxifraga fortunei* var. *incisolobata*	F35	24	Hakusan City, Ishikawa Pref., Japan	36°11′N, 136°36′E	KYO_00025616
*Saxifraga fortunei* var. *incisolobata*	F38	24	Sakai City, Fukui Pref., Japan	36°08′N, 136°22′E	KYO_00025339
*Saxifraga fortunei* var. *obtusocuneata* (Makino) Nakai	F67	24	Niyodogawa‐cho, Agawa‐gun, Kochi Pref., Japan	33°39′N, 133°08′E	KYO_00025613
*Saxifraga fortunei* var. *suwoensis* Nakai	F75	24	Imari City, Saga Pref., Japan	33°13′N, 129°53′E	KYO_00025356
*Saxifraga acerifolia* Wakabayashi & Satomi	SAF	24	Sakai City, Fukui Pref., Japan	36°08′N, 136°22′E	KYO_00025333

Note

*N* = number of individuals.

Vouchers are deposited at Kyoto University (KYO), Kyoto, Japan.

## Characteristics of 21 monomorphic microsatellite loci developed in *Saxifraga fortunei* var. *incisolobata*.^a^

**Table:**

Locus	Primer sequences (5′–3′)	Repeat motif	Allele size range (bp)	BLASTX top hit description	*E*‐value	GenBank accession no.
SF230	F: CACGACGTTGTAAAACGACCTGATTGCGACGATGAGAGC	(AT)_14_	413	No significant hit	—	LC465770
	R: GTTTCTTGTGCCTAACTTTCACCAACCC					
SF1095	F: CTATAGGGCACGCGTGGTTTTGAACGCCTTAAGACCGC	(AT)_18_	448	Probable E3 ubiquitin‐protein ligase BAH1‐like [*Herrania umbratica*]	7.92E‐41	LC465772
	R: GTTTCTTCGCTCGCCTTACTATAACCG					
SF561	F: CTATAGGGCACGCGTGGTGATTTGGAGCCTCTTTGCCG	(AT)_11_	317	No significant hit	—	LC465778
	R: GTTTCTTTTGACACCAGCCCTCACTAG					
SF293	F: CTATAGGGCACGCGTGGTAAACGAGACATGGCTGCTTG	(TTG)_6_	215	No significant hit	—	LC465781
	R: GTTTCTTTCGGGTTTGGTCACAGAGAG					
SF112	F: CGGAGAGCCGAGAGGTGTTTGAGAGTGGGCTGCCATC	(AT)_12_	135	Sphinganine C4‐monooxygenase [*Actinidia chinensis* var. *chinensis*]	1.66E‐164	LC465785
	R: GTTTCTTCGTGGTGCTATGTGACTTGG					
SF1055	F: CTATAGGGCACGCGTGGTGAGTAAGAGGTGGTGGAAACG	(AG)_12_	153	Stomatal closure‐related actin‐binding protein 1‐like [*Ziziphus jujuba*]	6.23E‐08	LC465787
	R: GTTTCTTATGCAAATCTCCTGGCAAGC					
SF785	F: CACGACGTTGTAAAACGACTCTTCTCAACGCTTGGTCTG	(ATC)_8_	152	PREDICTED: probable acyl‐activating enzyme 16, chloroplastic [*Populus euphratica*]	0.00	LC465789
	R: GTTTCTTTCGCGTGAGATCCAACATTG					
SF1547	F: CTATAGGGCACGCGTGGTAGGCGACGTGTCAGAGTATC	(AT)_10_	454	Hypothetical protein CDL15_Pgr014134 [*Punica granatum*]	5.14E‐06	LC465795
	R: GTTTCTTGAAGAAGCTCGTGATCAGGC					
SF1009	F: CGGAGAGCCGAGAGGTGAACCCATCTACTAGCAGGCG	(CAC)_8_	445	Trihelix transcription factor GTL1 isoform X2 [*Rosa chinensis*]	4.73E‐95	LC465796
	R: GTTTCTTGTTGTGGCTGTACTTGTGGC					
SF795	F: CTATAGGGCACGCGTGGTGACCGCCCTTTACCTTGTTG	(ATC)_7_	435	Uncharacterized protein LOC110645629 [*Hevea brasiliensis*]	1.26E‐136	LC465797
	R: GTTTCTTACAGAGAAGCATCCAGACCC					
SF1496	F: CTATAGGGCACGCGTGGTAGGCGGCTAAGATTGAGGAG	(AAG)_7_	445	Dehydrin [*Corchorus capsularis*]	4.00E‐03	LC465798
	R: GTTTCTTGTGGTGGAGGAGGAGTACAC					
SF145	F: CTATAGGGCACGCGTGGTTATCCCAAAGCAGCAGGAGG	(CCT)_7_	318	PREDICTED: pentatricopeptide repeat‐containing protein At4g38150‐like [*Camelina sativa*]	6.69E‐29	LC465800
	R: GTTTCTTAGGATTGGTTGAGGGAGACG					
SF316	F: CGGAGAGCCGAGAGGTGTGGGACGATACTTCACCGAC	(CT)_10_	344	Lectin_legB domain‐containing protein [*Cephalotus follicularis*]	5.08E‐51	LC465801
	R: GTTTCTTGGCCATGGATGAGGTGAAAC					
SF111	F: CACGACGTTGTAAAACGACGCCAGTCCAATAAGTTCGGC	(AT)_11_	307	PREDICTED: protein NLRC3 [*Prunus mume*]	1.37E‐101	LC465802
	R: GTTTCTTCCTGCAATGGAGTGACTGAAC					
SF1530	F: TGTGGAATTGTGAGCGGCGGTGAGAACGGAACAATGG	(ATC)_7_	279	Zinc‐finger homeodomain protein 5 [*Jatropha curcas*]	3.15E‐08	LC465803
	R: GTTTCTTTTGAGGATTCTGTGCCTCCG					
SF1096	F: CTATAGGGCACGCGTGGTTTCGACAGCAAACCGTTAGC	(TGG)_8_	339	PREDICTED: formin‐like protein 1 [*Vitis vinifera*]	0.00	LC465806
	R: GTTTCTTATTATCCGGCTCCATCTCGG					
SF553	F: CACGACGTTGTAAAACGACCGCAGAGGAGTTTACGCTTG	(TCG)_7_	271	PREDICTED: calcium‐dependent protein kinase 11‐like [*Juglans regia*]	0.00	LC465808
	R: GTTTCTTGTCATCGTCACAATCAACCAC					
SF1204	F: TGTGGAATTGTGAGCGGACCACAAACGTATCTAGGCATG	(AGC)_7_	245	Beta‐amyrin 28‐oxidase‐like [*Quercus suber*]	5.67E‐95	LC465809
	R: GTTTCTTAACGACCCAAACAAGCACAG					
SF76	F: CGGAGAGCCGAGAGGTGCCATTCTCGCTCCAACATCG	(ACC)_7_	286	SANT/Myb domain [*Macleaya cordata*]	2.19E‐81	LC465810
	R: GTTTCTTGGCGCTGGAGGATTAGAATG					
SF1403	F: CTATAGGGCACGCGTGGTCATCCGCCAGCATGTGATG	(TC)_17_	169	PREDICTED: trigger factor‐like protein TIG, Chloroplastic [*Populus euphratica*]	0.00	LC465811
	R: GTTTCTTGCAGCTAGTGAAGTGATGGAG					
SF1504	F: CGGAGAGCCGAGAGGTGAGCGTGACCTTAACCTCCTC	(ATC)_7_	179	Membrane‐associated kinase regulator [*Actinidia chinensis* var. *chinensis*]	9.89E‐46	LC465812
	R: GTTTCTTGTCCGAGGAAGACGAAGGAC					

a Annealing temperature is 58°C for all primer pairs.
